# A Comparative Study on the Amount of Extruded Material from the Apical Foramen with NiTi Rotary and Stainless Steel Hand Instruments

**Published:** 2006-07-01

**Authors:** Shahrzad Nazari, Farshid MirMotalebi

**Affiliations:** 1 *Department of Endodontic, Dental School, Hamedan University of Medical Sciences, Hamedan, Iran*; 2 *Department of Prosthodontic, Dental School, Hamedan University of Medical Sciences, Hamedan, Iran*

**Keywords:** Extruded Debris, Root Canal Therapy, Rotary Instruments

## Abstract

**INTRODUCTION:** The purpose of the present study was to quantify and compare the amount of extruded debris from apical foramen after instrumentation of the root canal system with hand and rotary instruments.

**MATERIALS AND METHODS:** Root canals of forty five fresh extracted single rooted human teeth with mature apexes and less than 15 degree of root curvature were instrumented in group A with stainless steel K-Type files, in group B with rotary NiTi Flex Master files, and in group C with rotary NiTi ProTaper files and followed weighting the extruded debris by a digital scale to within 0.0001 gram accuracy.

**RESULTS:** In all groups, the mean weight of extruded debris was not more than 5 mg (P=0.0l) and was ranked as: Group A>Group B>Group C. There were statistically significant differences among three groups (p=0.0l). The mean value of extruded debris in the ProTaper and Flex Master groups were 0.652 and 0.788 mg, respectively.

**CONCLUSION:** The study revealed that the amount of extruded debris from the apical foramen was minimal when ProTaper files were used.

## INTRODUCTION

Flare-Up during endodontic treatment has been a persistent problem for years (1). Along root canal therapy, new 1rntants such as medications, cleaning solutions or modified proteins may enter into granulomatous lesion. Flare-Up is multi-factorial with varied symptoms (2).The causative factors of flare­ ups comprise mechanical, chemical and/or microbial injury of the pulp or periradicular tissues. There are some situations during endodontic therapy in which balance between microbial aggression and host defenses may be disrupted in favor of microbes and an acute periradicular inflammation can ensue (3).It has been shown in a clinical trial that apical instrumentation and overfilling can lead to both significantly higher incidence of resistant periapical lesion and lower incidence of complete remedy (4). From these, we can mention apical extrusion of infected debris in the most. Based on these situations, preventive measures against infective flare-ups are proposed and selection of instrumentation technique that extrudes lesser amounts of debris apically is the first (3).

Debris extrusion from apical foramen is a sequel for all instrumentation techniques, but some methods provide less. In contrast, hand instrumentation has been shown to extrude more debris. Besides, coronal canal preparation before apical cleaning may reduce this side­ effect (4-16). Following NiTi rotary system introduction vanous studies have been accomplished to calculate the amount of extruded debris from apical foramen in comparison to Stainless Steel (SS) hand files.

McKendry in 1990 compared Balanced forces technique (BF) with Endosonic and hand instrumentation with step-back technique and concluded that the BF technique extruded less debris apically than either Endosonic or step­ back techniques (5). In 1991 Montgomery and Myers compared a rotary instrumentation system (Canal Master) to hand filing method. They concluded that filing short of AF can cause apical plug formation which inhibits debris extrusion (6). In 1995, Al-Omary and Dummer showed that among eight hand instrumentation methods, step back caused the highest amount of debris compared to Balanced Force and Crown Down Pressure-less technique (7). Hartwell and Beeson in 1998 emphasized on the precise control of working length. In their study, step back (SB) hand filing was compared to rotary ProFile taper 0.04 in regard to amount of extruded debris. They concluded that ProFile taper 0.04 files (canal preparation l mm short of WL) caused the least debris beyond apical foramen despite the technique (8). In 1998, Reddy and Hicks investigated the quantity of apical debris produced In Vitro using two hand and Rotary systems. They showed that there was no difference between balanced forces, Lightspeed and ProFile with respect to total extruded debris (9). Hinrichs, walker and schindler in 1998 compared the amount of apically extruded debris using Lightspeed, ProFile 0.04 taper series 29, and NT McXIM Ni-Ti engine driven instruments and flex-R files in BF technique and noticed that there was no statistically significant differences among four groups (10). In 2000, Hulsman et al. performed a study comparing canal preparation by Quantec, ProFile taper 0.04, Light speed and Hero 642 systems. They stated that Hero 642 caused the least amount of extruded debris followed by Quantec, LightSpeed and ProFile (11). Ferraz et al. in their study compared two hand and three Rotary instruments with respect to amount of apical extrusion pf debris. These researchers concluded that there were no statistical differences between the BF technique and the engine-driven methods (12). In an In vitro study, the effect of maintaining apical patency on periapical extrusion was determined by Lambriandis, Tzoanopoulou and Tosounidou. They showed that there were significant differences in the amount of extruded material before and after the enlargement of the apical constriction with greater extrusion when the constriction remained intact (13).

Bidar et al. in 2004 evaluated the amount of apically extruded debris in conventional and rotary instrumentation techniques. They mentioned that the differences in the amount of debris produced among Rotary groups (ProFile 0.04 taper series Rotary system at three speed: 1000, 2000 and 24000 rpm) was not significant (14). In 2005, Azar and Ebrahimi conducted a comparative investigation on the amount of apically extruded debris using the ProTaper, ProFile and Hand instrumentation techniques. They showed that although the mean amount of extrusion with the step-back technique was higher than the two Rotary systems, there were no significant differences between the three groups (15).

The purpose of this study was a comparative evaluation of extruded debris out of apical foramen by rotary NiTi and hand stainless steel files dung routine endodontic treatment.

## MATERIALS AND METHODS

45 recently extracted human single rooted teeth were included in the study. Inclusive criteria were based on the presence of a non­ obliterated canal, fully developed apex and root curvature less than 30°, which was determined by radiographic and visual examination. Soft tissue and calculus removal was carried out mechanically and samples were immersed in 5.25% sodium hypochlorite for 48 hours. For maximum homogeneity of samples, crowns were cut by diamond disks and final 19 mm length was assigned to the remaining root. Which were then conserved in 9% normal saline until studied. For reducing inter operator errors, all procedures were done by single operator. Forty five teeth were randomly divided into three groups as follows: group A: Instrumentation by K-type Hand files (Maillefer, Dentsply, Switzerland), group B: Instrumentation by rotary Flex Master files (VDW, Germany), and group C: Instrumentation by rotary ProTaper files (Tulsa dental, Tulsa, OK, USA).

According to the method described by Montgomery and Myers, extruded debris from apical foramen during canal instrumentation was collected in vials containing distilled water, which were used as a collector for debris. The vials were then put in glass flasks in order to be protected from dust. Before mounting the roots, these clean and dry flasks were weighed by a digital balance to 0.0001 gram accuracy level followed by encoding and filling it with 3cc distilled water.

In all groups, canal instrumentation was carried out 1 mm short of the apical foramen. The total volume of irrigating solutions (2.6% sodium hypochlorite) was 10 ml per canal, and was delivered through a 28-gauge endodontic irrigation needle. By using a rubber stopper, the depth of the needle insertion into the canal was set at 8 mm. Each 1 ml of irrigant solution was delivered in 10 sec.

In group A canal preparation was done with crown down Pressure less technique (CDPT) described by Marshall and Pappin with S.S. hand files so that # 30 file was passed easily up to working length. In group B after performing coronal preparation with IntroFile, the filing sequence was done according to the manufacturerʼs instruction. Apical enlargement was done by blue # 30 files with 0.2 and 0.04 taper. In group C coronal canal preparation was done by Shaper X with 8 mm working length. Then canal preparation was completed by finishing files in a way that those files with 0.03 tip diameters were passed to working length. After that the roots were carefully pulled out of the lid and the perforated lid was replaced by a new one. Flasks containing vials were weighed again by the aforementioned balance and considering the vial codes, the results obtained were compared to those of preoperative values.

Weight difference was determined by reducing the amount of extruded materials which consisting necrotic debris and irrigants after breaking the codes, data were analyzed by one way ANOVA and post hoc test and p value was adjusted at 0.01.

**Table 1:** The amount of extruded debris during endodontic treatment by hand and rotary files in milligrams

**Group**	**A**	**B**	**C**
Mean	1.18	0.788	0.652
Standard Deviation	1.04	0.821	0.674
Standard Error	0.73	0.27	0.20
Maximum	2.70	1.43	1.61
Minimum	0.05	0.02	0.01

## RESULTS

According to the data demonstrated in Table 1 the least amount of apically extruded debris is produced by Rotary ProTaper system. Data analysis by tests of ANOVA and Tukey HSD as a post hoc test showed significant differences between hand and Flex Master or ProTaper, in addition to between Flex Master and ProTaper (Table 2). Maximum and minimum values of extruded debris belong to hand files (2.7 mg) and ProTaper Rotary system (0.01 mg), respectively.

## DISCUSSION

This study was done in order to evaluate and compare the efficacy of rotary versus hand instrumentation techniques. Sample size was determined based on similar studies. Sample selection was performed strictly according to inclusive criteria to both maximize the validity of the results and avoid interference of intervening variables.

All procedures were done by a single operator. Single rooted teeth were included of variables. No attempt was made to differentiate the teeth based on jaws. Clinically, extreme curvatured roots were excluded. After decoronation, roots were adjusted at 19 mm to have a reliable reference point for working length determination, a better visualization of canal morphology and finally to avoid coronal interferences during early preparation (9).

According to Schneider method introduced in 1997, pre operative radiographs were evaluated to exclude samples having root curvature of more than 30° (6).

Thus, by using this standardized tooth model in the current study, the amounts of collected extruded materials was mostly a result of instrumentation differences, and not the tooth morphology or inter operative errors (13).

**Table 2:** Statistical analysis (*One-way ANOVA and post hoc test*) of the amount of extruded debris for three filing systems

**Group**	**No**	**extruded debris**	**F**	**p ** **value**
A	15	1.18± 1.04 (mg)	**F=21.27**	0.01^a^
B	15	0.788±0.821		0.01^a^
C	15	0.652±0.674		0.01^a^

^a^: *Significant*

Some previous studies did not measure the amount of extruded irrigants (16) so in the present study we tried to simulate the clinical conditions in which both debris and irrigants were responsible for post operative flare-ups (1,2). Therefore the total amount of materials extruded from the apical foramen was collected and weighted (14).

In order to minimize the variables during the irrigation process, equal amount of irrigation solution was used and the procedure was applied by controlled forces in 10 sec (14).

Based on the result of this study, Rotary ProTaper files provided the least amount of extruded debris during routine endodontic treatments followed by Flex Master. On the contrary, hand stainless steel files (k-type) produced the highest value.

Finally we concluded from the present study that no technique can inhibit debris extrusionfrom apical foramen; although it can be minimized by utilizing some special hand instrumentation techniques or rotary systems along with exact control of working length.
